# Emphysematous Aortitis due to *Clostridium septicum* in an 89-Year-Old Female with Ileus

**DOI:** 10.1155/2019/1094837

**Published:** 2019-08-27

**Authors:** Susana Urgiles, Harold Matos-Casano, Kyaw Zin Win, Jeronimo Berardo, Utpal Bhatt, Jilan Shah

**Affiliations:** ^1^Department of Internal Medicine, Wyckoff Heights Medical Center, 374 Stockholm Street, Brooklyn, NY 11237, USA; ^2^Division of Pulmonary Disease and Critical Care Medicine, Wyckoff Heights Medical Center, 374 Stockholm Street, Brooklyn, NY 11237, USA; ^3^Division of Infectious Disease, Wyckoff Heights Medical Center, 374 Stockholm Street, Brooklyn, NY 11237, USA

## Abstract

Emphysematous aortitis is a rare but lethal form of infectious vasculitis. This condition was found incidentally on computed tomography of the chest during the evaluation of a patient presenting with pneumonia coincident with adynamic ileus. The patient did not have a history of malignancy. While colon cancer could not be ruled out, it is possible that ileus may have contributed to or resulted in bacterial translocation in this case. Appropriate investigations and empirical therapy against *Clostridium septicum* should be initiated in the presence of clinical and radiological findings suggestive of emphysematous aortitis.

## 1. Introduction

Emphysematous aortitis (EA) is a rare form of vasculitis with a nonspecific clinical presentation and high fatality rates. Malignancy, which is a frequent finding in these patients, causes loss of integrity of the intestinal mucosa, facilitating *Clostridium septicum* (CS) bacteremia and infection at distant sites. Adynamic ileus may also cause bacteremia through similar mechanisms. This observation underscores the need to consider other less common mechanisms of bacterial translocation during the assessment of EA due to CS.

## 2. Case Description

An 89-year-old female was admitted to our institution after three days of subjective fevers, dyspnea, productive cough, malaise, and anorexia. She denied having chest or back pain. Medical history included hypertension, hyperlipidemia, type 2 diabetes mellitus, and stage 4 chronic kidney disease, with no history of surgical interventions or toxic habits. Diverticulosis and a 6 mm sigmoid hyperplastic polyp which was completely removed were identified on colonoscopy five years earlier.

On exam, the patient's temperature was 99.0°F, heart rate 108/min, respiratory rate 30/min, blood pressure 148/100 mmHg, and oxygen saturation 92% on 2 liters of oxygen via nasal cannula. She appeared ill. Bilateral inspiratory crackles and decreased breath sounds at the left lung base were auscultated. Her abdomen was distended, and bowel sounds were absent. The physical exam was otherwise unremarkable.

**Figure 1:**
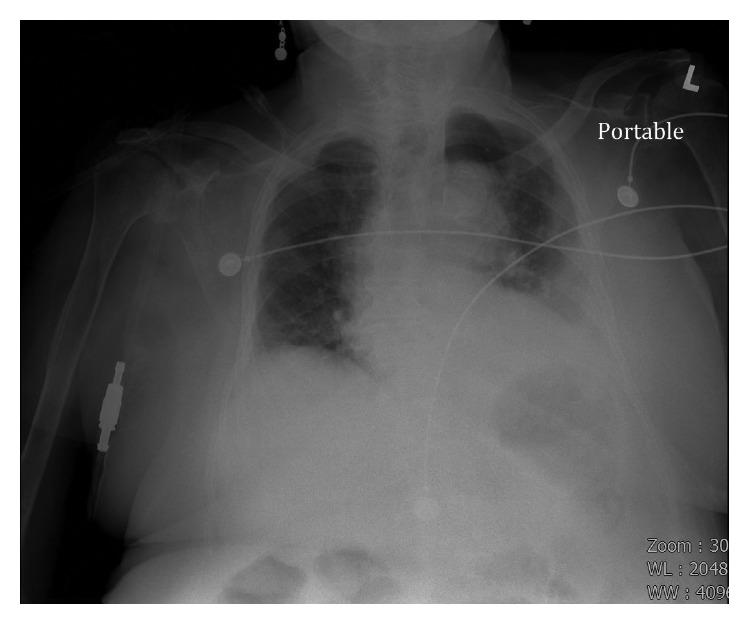
Chest X-ray obtained on admission showing left lower lung infiltrate, with blunting of the adjacent costophrenic angle consistent with a pleural effusion.

**Figure 2:**
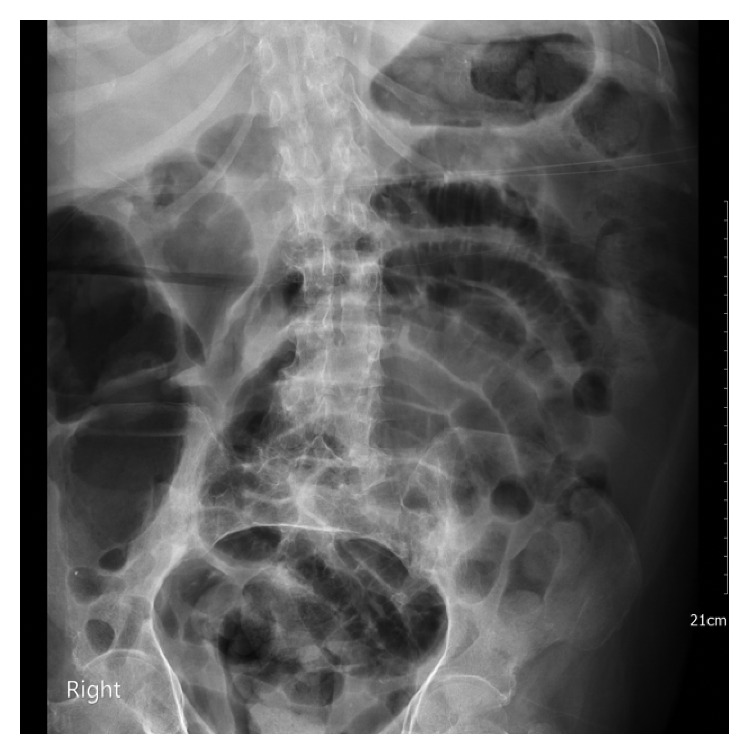
Diffuse gaseous distention of the bowel consistent with ileus seen on abdominal X-ray.

**Figure 3:**
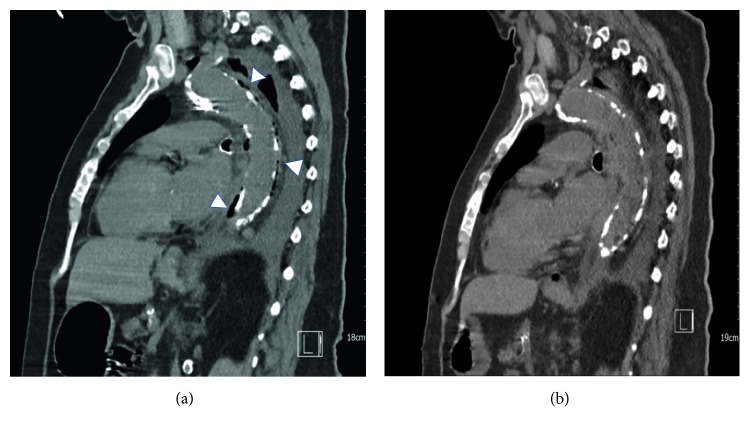
Sagittal view on chest CT obtained on admission (a). Note the presence of periaortic gas and inflammatory thickening of the aortic wall (arrowheads) without aneurysmal dilation, extending along the aortic arch and descending aorta. By admission day 10, CT findings showed resolution of the periaortic emphysema with interval development of nonspecific fluid and inflammatory changes (b).

Significant laboratory findings included leukocytosis of 26.8 K/*μ*L with neutrophilia (81%), creatinine of 3.32 mg/dL, and normal lactic acid at 1.5 mmol/L. Fecal occult blood testing was negative. A chest radiograph revealed a tortuous, calcified aorta and a left pleural effusion with adjacent infiltrates (Figure 1). Abdominal X-ray revealed diffuse gaseous distension of the bowel consistent with ileus (Figure 2). Abdominal ultrasound was significant only for multiple gallstones. The visualized abdominal aorta was unremarkable. No suspicious bowel lesions were observed. Computed tomography (CT) of the chest revealed left lung infiltrates and a moderate left pleural effusion. Incidentally, numerous foci of soft tissue gas were noted within the aortic wall, extending from the arch to the distal descending aorta (Figure 3(a)). Treatment for emphysematous aortitis and pneumonia in a patient with penicillin allergy was initiated with intravenous vancomycin, aztreonam, and clindamycin, especially to cover anaerobes such as clostridial species.

Cardiothoracic surgery consultation was obtained. However, the patient was not considered a surgical candidate since she was clinically unstable, having developed atrial fibrillation with rapid ventricular response and hypertensive crisis at this time, requiring transition of care to the intensive care unit.

The patient was eventually stabilized and transitioned to the general medicine ward. CS was subsequently isolated in the first three consecutive anaerobic blood culture bottles. Pleural fluid obtained five days after admission was exudative, with pleural fluid to serum protein ratio greater than 0.5 and pleural fluid to serum lactate dehydrogenase (LDH) ratio greater than 0.6 (pleural fluid: glucose 159 mg/dL, total protein 3.7 g/dL, and LDH 258 IU/L; plasma: glucose 127 mg/dL, total protein 5.9 g/dL, and LDH 312 IU/L). Pleural fluid cultures were negative. On admission day 10, a follow-up chest CT showed fluid and inflammatory changes replacing the gas pockets seen on the initial study (Figure 3(b)). CT imaging of the abdomen with intravenous contrast was considered as a noninvasive alternative to evaluate the presence of any intra-abdominal malignancy since the patient was not stable to undergo colonoscopy. However, the patient's renal function precluded the use of this imaging modality. Upon discussion of the condition, its management, and prognosis with the patient and her healthcare proxy, the decision was made to transition the patient's care to hospice. Colonoscopy was therefore not pursued, and after 12 days of intravenous antibiotics, a 6-week course of oral clindamycin was prescribed at the time of discharge. It has not been possible to confirm whether and for how long the patient survived after this.

## 3. Discussion

CS is an opportunistic, Gram-positive, spore-forming anaerobe, found in any habitat with organic compounds. Notably, it causes atraumatic gas gangrene with myonecrosis resulting from the release of hyaluronidase, hemolysin, lethal necrotizing and other toxins [1]. Occurrence of spontaneous gas gangrene by CS is usually tied to conditions leading to either enhanced bacterial invasiveness, increased host vulnerability to infection, or both. Mucosal changes observed in colitis and colon cancer result in low pH, hypoxia, and increased lactate concentrations in affected tissue [2, 3]. CS spores proliferate under these conditions, which are also conducive to the development of intestinal ulcerations through which CS may enter the bloodstream [4]. Malignancy and diabetes are, respectively, the first and second most frequent conditions associated with atraumatic gas gangrene [5]. Overall, CS infections are unusual, representing approximately 1.3% of all caused by Clostridia [6].

Infectious aortitis is a rare entity, accounting for 2.6% of aortic aneurysms [7]. CS is the most common causal agent. Risk factors include a history of prior vascular instrumentation, atherosclerosis, and colon cancer, which has been reported in 82.5% of cases [5]. In other cases, EA has been associated with Crohn's disease [8], ulcerative colitis [9], diverticulosis [10], and benign polyps [11]. It has also been observed in patients without neoplastic disease including young, otherwise healthy individuals [12–15]. CS-relative aerotolerance facilitates its ability to invade well-oxygenated tissue, leading to the rapid development of an infected aneurysm [1]. Clinical features are often nonspecific and include fever, chills, and pain localized to the chest, back, or abdomen [7]. Early diagnosis can be usually made with CT, which shows periaortic gas in 92.6% of cases. Treatment involves surgery and intravenous antibiotics. In cases where surgery is performed electively, a course of antibiotics precedes the intervention, which is then followed by at least six weeks of intravenous antibiotics. This course could then be followed by life-long oral-suppressive antibiotic therapy [5]. Antibiotics should be initiated as soon as suspicious lesions are identified with high-dose penicillin plus clindamycin, which is thought to prevent toxin synthesis [16]. Metronidazole and vancomycin could be used as alternative agents in those allergic to penicillin [1]. The overall 6-month mortality associated with CS EA is 79.5%, which increases to 100% if no surgery is performed [5].

Contrasting with most cases, our patient had no history of aortic instrumentation. The case also highlights the utility of CT in early diagnosis since periaortic gas was visualized days before CS was isolated. Since the patient had two distinct infectious processes and a history of penicillin allergy, antibiotic selection was guided by the need to cover against anaerobic, Gram-positive, and Gram-negative bacteria.

The inability to exclude the presence of gastrointestinal malignancy represents a limitation of this case study. As indicated in the case description, the patient's critical condition at presentation, renal failure, and subsequent request for the provision of hospice care precluded further investigations in this regard. A review of the patient's health records showed that no suspicious masses were found on noncontrast CT studies of the abdomen and pelvis obtained within two years prior to this admission. Regarding her colonoscopy findings, it appears that small rectosigmoid hyperplastic polyps are not associated with an increased risk of colorectal cancer [17, 18], and guidelines recommend surveillance colonoscopy in 10 years from the baseline study [19]. While malignancy could not be conclusively ruled out, the above considerations, together with the absence of weight loss, anorexia, a negative fecal occult blood testing at presentation, and the nonvisualization of sizable intestinal masses on ultrasound, may suggest the presence of colorectal cancer was less likely. As noted above, both diverticula and benign polyps, which were found on the patient's colonoscopy, have been associated with EA and could have contributed to the occurrence of bacteremia in this case. In addition, the patient's diabetes and extensive atherosclerosis are by themselves significant risk factors associated with EA.

An interesting consideration in this case concerns the potential association between CS bacteremia and adynamic ileus, which may have resulted from the left lower lobe pneumonia. As seen with colitis and malignancy, ileus can induce local ischemia-reperfusion injury, microvascular thrombosis, and inflammatory changes causing mucosal ulcerations, damage to the mucosal-associated lymphoid tissue, and impairment of other local protective mechanisms [20]. In addition, bacterial overgrowth leads to intraluminal nutrient depletion. The resultant dysbiosis eliminates the protective role of the normal intestinal flora, with a predominance of anaerobes such as *Bacteroides* and *Clostridium*. Finally, pathogenic virulence factors activate local and systemic inflammatory responses, affecting host immunity and giving way to bacterial translocation into the lymphatic and venous circulation through mucosal defects [20–25].

## 4. Conclusion

CS aortitis is rare and lethal. While its clinical presentation is usually nonspecific, emphysematous aortitis should be included in the differential diagnosis of patients presenting with systemic signs of infection and nonspecific chest or back pain, abdominal discomfort, and a history of aortic atherosclerosis, diabetes, and/or colon cancer. In their assessment, clinicians should be alert to the possibility that other conditions such as colitis, diverticulosis, benign polyps, and possibly ileus may create lesions serving as potential ports for bacteria to enter the circulation. Finally, considering that an aneurysm may appear only at more advanced stages of the infection, it would be reasonable to evaluate the aorta with CT imaging upon isolation of CS in blood cultures of otherwise asymptomatic patients, to rule out the presence of incipient EA.
